# Modification of Tumor Necrosis Factor-α and C-C Motif Chemokine Ligand 18 Secretion by Monocytes Derived from Patients with Diabetic Foot Syndrome

**DOI:** 10.3390/biology9010003

**Published:** 2019-12-22

**Authors:** Karine O. Galstyan, Ludmila V. Nedosugova, Narine S. Martirosian, Nikita G. Nikiforov, Natalia V. Elizova, Kira I. Kolmychkova, Igor A. Sobenin, Alexander N. Orekhov

**Affiliations:** 1 Federal State Autonomous Educational Institution of Higher Education I.M. Sechenov First Moscow State Medical University (Sechenov University), Ministry of Health of the Russian Federation, 119991 Moscow, Russia; profmila@mail.ru (L.V.N.); narinarine@list.ru (N.S.M.); 2 City Clinical Hospital № 67 named after L.A. Vorokhobov, 123423 Moscow, Russia; 3 Laboratory of Medical Genetics, Institute of Experimental Cardiology, National Medical Research Center of Cardiology, 121552 Moscow, Russia; nikiforov.mipt@googlemail.com (N.G.N.); igor.sobenin@gmail.com (I.A.S.); 4 Laboratory of Angiopathology, Institute of General Pathology and Pathophysiology, 125315 Moscow, Russia; niiopp@mail.ru (N.V.E.); kirruccha@rambler.ru (K.I.K.); a.h.opexob@gmail.com (A.N.O.); 5 Center of Collective Usage, Institute of Gene Biology, 119991 Moscow, Russia; 6 Research Institute of Threpsology and Healthy Longevity, Plekhanov Russian University of Economics, 117997 Moscow, Russia

**Keywords:** type 2 diabetes mellitus, diabetic foot syndrome, monocyte response, pro-inflammatory, cytokine, anti-inflammatory, chemokine, tumor necrosis factor-α, C-C motif chemokine ligand 18

## Abstract

**Background:** This study involves the investigation of spontaneous and induced secretion of the pro-inflammatory cytokine tumor necrosis factor-α (TNF-α) and the anti-inflammatory chemokine C-C motif chemokine ligand 18 (CCL18) by monocytes isolated from blood of patients with long-term type 2 diabetes mellitus (T2DM), both with or without foot ulcers. **Methods:** A total of 121 patients with T2DM (79 without diabetic foot syndrome (DFS) and 42 patients with DFS) were included. Cluster of Differentiation 14 (CD14+) monocytes were isolated from patients’ blood and stimulated by interferon-γ (IFN-γ) and interleukin-4 (IL-4) for induction of pro- and anti-inflammatory monocyte activation, respectively. The concentrations of TNF-α and CCL18 in the culture medium were measured using ELISA on day 1 and day 6 after cell stimulation. **Results:** We found a correlation between glycated hemoglobin (HbA1c) and stimulated secretion levels of TNF-α (*r* = 0.726, *p* = 0.027) and CCL18 (*r* = –0.949, *p* = 0.051) in patients with DFS. There was an increase of pro- and anti-inflammatory activation of monocytes in all patients with different durations of DFS (*p* < 0.05). However, no stimulation of anti-inflammatory activation was detected in patients with DFS lasting more than 6 months (*p* = 0.033). **Conclusions:** Our study showed an increase in pro-inflammatory secretion and a decrease in anti-inflammatory secretion by monocytes isolated from blood of patients with T2DM depending on HbA1c levels and duration of the inflammatory process. These findings allow us to assume that monocytes isolated from T2DM patients are characterized by a biased ability to respond towards pro-inflammatory stimulation, contributing to the chronic wound process.

## 1. Introduction

Diabetes mellitus (DM) is an endocrine disease characterized by chronic hyperglycemia. According to the National Register (Gosregistr) of DM, there were 3,988,718 patients suffering from type 2 diabetes mellitus (T2DM) in Russia from 2000 to 2015. However, the results of the National epidemiological observational cross-sectional study (NATION study) (2016) indicate that only about 50% of DM patients are diagnosed [1]. According to World Health Organization (WHO) data, the number of patients with DM was 174 million individuals worldwide in 2015, reaching 236 million a year later [2]. The number of patients with late complications of DM expectedly increases. Among numerous complications of DM, there is damage to the peripheral nervous system in the setting of hyperglycemia, resulting in irreversible changes leading to micro- and macrovascular disease [3]. The most common cause of adverse effects on the nervous system is long-term metabolic imbalance [4]. Various clinical studies confirm this fact. Diabetic neuropathies and angiopathies also cause cell dysfunction and impaired homeostasis of tissues and whole organs [5]. In cases of insulin deficiency, the need for glucose in nerve tissue activates the polyol pathway and leads to the development of oxidative stress through excessive production of free radicals [6]. In the setting of hyperglycemia, atherosclerosis progresses as a result of oxidative modification of low density lipoproteins (LDL) followed by enhanced absorption by monocytes, which are transformed into foam cells involved in the pre-atherogenic lipid infiltration of the vascular wall [7,8]. Modified LDL particles begin to aggregate spontaneously following a change in the surface charge [9]. The presence of inflammatory mediators such as tumor necrosis factor-α (TNF-α) and interleukins (IL) in atherosclerotic plaque supports the chronic inflammation [10]. Signs of local and systemic nonspecific inflammatory processes in atherosclerosis emerge at initial stages of lesion in the artery wall [11,12]. Blood cells such as monocytes play a key role in the process of atherosclerotic inflammation. Monocytes, while migrating into the sub endothelial space, are differentiated into macrophages involving many transcription factors, with the nuclear transcription factor kappa B (NF-κB) considered as the major one. Cytokines and inflammatory mediators activate NF-kB in neurons and neuroglia, promoting nerve proliferation [13]. Activated NF-kB during oxidative stress is responsible for expression of 500 different genes, enzymes, and pro- and anti-inflammatory mediators. Expression of NF-kB is most pronounced in patients with chronic diseases, including patients with diabetes. Activation of NF-κB in DM is associated with glucose self-oxidation and excessive formation of reactive oxygen species (ROS), leading to activation of protein kinase C [14]. Monocytes that penetrate into the intima partially go through differentiation and proliferation, turning into macrophages under the influence of colony-stimulating factors Macrophage colony-stimulating factor (M-CSF), Granulocyte-macrophage colony-stimulating factor (GM-CSF) and other factors secreted by endothelial cells. They express scavenger receptors, and then transform into foamy cells. The flexibility of monocytes is associated with their transformation into pro-inflammatory and anti-inflammatory responses. M-CSF induces formation of a monocyte phenotype that does not transform into foam cells and further secretes pro-inflammatory cytokines such as IL-1, IL-6, IL-8, IL-12, and TNF-α. This monocyte transformation pathway is defined as the classical pro-inflammatory activation pathway [15]. An anti-inflammatory pathway is activated by GM-CSF. In this pathway, modified monocytes acquire the ability to secrete anti-inflammatory cytokines and chemokines, i.e., IL-4, IL-10 IL-13, Transforming growth factor—beta (TGF-β), C-C motif chemokine ligand 18 (CCL-18), etc. This alternative transformation pathway provides control over the all chronic inflammatory processes. Under normal conditions, monocytes are inactive. The pro-inflammatory phenotype is activated in the presence of intracellular pathogens or under the influence of interferon-γ (IFN-γ), while the anti-inflammatory phenotype is stimulated by extracellular parasites or IL-4. Their ability for activation is assessed by secreted cytokine and chemokine levels. Monocytes can be activated in an inflammatory environment, and this process depends on the microenvironment. Dysregulated monocytes control the development of inflammatory diseases [16]. With the development of regenerative medicine and tissue engineering, the role of monocytes and the cytokines and chemokines secreted by them has become critical [17].

Monocyte dysfunction is associated with the origin and development of autoimmune diseases, neoplasms, and other chronic inflammatory conditions [18]. Recent studies have shown the role of molecular mechanisms in the development of DM. These involve regulation and transport of peptides, as well as control of the trend and intensity of the immune attack [19]. DM is associated with a chronic inflammatory condition that ultimately leads to imbalance and impaired regulation of the skin immune function. Dysregulated production of Reactive oxygen species (ROS), Reactive Nitrogen (RN), and proteases leads to the persistence of inflammation. The diabetic wound environment is characterized by excessive and prolonged inflammation, which is associated with poor healing, and in humans leads to the development of diabetic foot ulcers. However, the underlying mechanisms that contribute to excessive inflammation remain uninvestigated.

The condition of the immune system may also contribute to the development of chronic wounds of the lower limbs. When wounds are healing properly, inflammatory cells initially show activation of both pro- and anti-inflammatory responses during the inflammatory phase (the first few days), and then move to the anti-inflammatory response during the proliferation/neovascularization healing phase (by day 7). The effect of the diabetic environment on the activation of inflammatory cells has also been recently studied. [20,21]. However, these studies did not consider the response of individual inflammatory cells in situ. Only limited data are available concerning regulation of pro- and anti-inflammatory monocytes activation and their performance in vivo. The question remains as to whether pro- and anti-inflammatory monocyte responses are predetermined in the tissue regenerative process or their differentiation depends on the local environment. Since pro- and anti-inflammatory processes are crucial in various phases of wound healing, one can assume that immune system disorders can interfere with tissue homeostasis and wound healing after the occurrence of foot ulcers and can lead to development of chronic, non-healing wounds that are characteristic of diabetic foot syndrome (DFS).

Therefore, the purpose of our study was to investigate the spontaneous and induced in vitro secretion of the pro-inflammatory cytokine TNF-α and the anti-inflammatory chemokine CCL18 by blood-derived monocytes from patients with long-term T2DM, both with and without DFS.

## 2. Materials and Methods

The study was conducted in 121 patients with T2DM. The first group included 28 patients with newly diagnosed T2DM not receiving glucose-lowering therapy at the time of hospitalization; the second group included 51 patients (time from DM diagnosis 10 years (5; 15)) with T2DM without DFS; and the third group included 42 patients with DFS (time from DM diagnosis 12 years (6; 20)). Patients of the second and the third groups received insulin therapy to achieve the target level of HbA1c and did not take metformin. Patients did not receive lipid-lowering drugs or other drugs that affect inflammation. All patients signed their informed consent for study participation before they enrolled in the study. The study was conducted in accordance with the Declaration of Helsinki, ICH GCP guidelines, and the protocol was approved by the Local Ethics Committee.

**Inclusion and Exclusion Criteria**

Main inclusion criteria were:

1. Signed and dated informed consent,
2. Male and female aged ≥ 35 years old, and
3. Patients with newly diagnosed T2DM not receiving glucose-lowering therapy, or
4. Patients suffering from T2DM without DFS, or
5. Patients suffering from T2DM with DFS, receiving insulin therapy.

Main exclusion criteria were:

1. Current inflammatory condition (odontogenic, pulmonary, pelvic, etc.) and/or chronic disease;
2. Current abuse of alcohol;
3. Current smoking;
4. Stage ≥ 3b chronic kidney disease including dialysis for acute renal failure within 12 months prior to inclusion in the study;
5. Acute Human Immunodeficiency Viruses (HIV) infection, hepatitis С/D virus, hepatic cirrhosis;
6. History of myocardial infarction and/or stroke within 2 months prior inclusion in the study;
7. Pregnancy or lactation;
8. Decompensated hypothyroidism;
9. Acute trauma, surgical or other condition;
10. Receiving metformin within 6 months prior to inclusion in the study;
11. Patients with foot ulcers with signs of a systemic inflammatory reaction.

Twenty-eight non-treated patients with newly diagnosed T2DM (11 male, 17 female) served as a control group [12]. Serum glycose levels were measured using the hexokinase method. Glycated hemoglobin (HbA1c) levels were assayed using the Capillarys 2 automated capillary electrophoresis system (SebiaSA, Evry, France), and the blood lipid profile was assayed using an automated biochemical analyzer AU 680 (Beckman Coulter, Brea, CA, USA). The dopplerometric blood circulation in lower limb arteries was measured with Smartdop 30 doppler analyzer, and the ankle–brachial index (ABI) was calculated. This parameter reflects the ratio of the systolic blood pressure (SBP) in the tibialis posterior to the SBP in the brachialis. Normally, the ABI value varies from 0.9 to 1.3. A value < 0.9 indicates stenotic lesions; and medial artery calcification develops at a value of > 1.3. We used quantitative tests to perform the neurological examination of the lower limbs. When included in the study, none of the patients showed clinical symptoms of systemic inflammation. Table 1 shows baseline clinical and laboratory parameters of the patients. There was no significant difference between groups of T2DM patients with different duration of the condition.

Data from patients from different groups were included in each series of cell isolation and cultivation, as well as measurements of cytokine secretion. In the current study we did not include a previously published comparative analysis of the activation abilities of monocytes isolated from the blood of healthy donors and T2DM patients, limited only by comparison of patients with T2DM with and without DFS [22]. Monocytes were derived from peripheral blood of patients using centrifugation with the density gradient of Ficoll followed by magnetic separation of CD14+ cells (Miltenyi Biotech, USA). Isolated cells were incubated over 24 h for plastic adhesion. Then, non-adherent cells were removed. This approach allowed achieving up to 95% viable monocytes. We did not reveal differences in the morphology of monocytes in patients from all groups. The remaining adherent cells were cultured in 24-well plates (1 × 10^6^ cells per well) in serum-free media X-VIVO 10 with 100 ng/mL of IFN-γ for 24 h or with 10 ng/mL of IL4 for 6 days. IFN-γ and IL4 were used for induction of pro- and anti-inflammatory activated monocytes as previously described [23]. After 1 and 6 days, cell viability did not decrease. Cytokine TNF-α was considered as a marker of pro-inflammatory activity of monocytes, while chemokine CCL18 used as a marker of anti-inflammatory activity. After incubation of monocytes with IFN-γ or IL4, the secretion levels of TNF-α and CCL18 were measured using ELISA.

The cytokine levels measured are expressed in pg/mL. Statistical analyses were performed using the 22.0 IBM SPSS Statistics and MS Excel 2013 software. Values are expressed as median (Me) and interquartile range (IQR) (Q25; Q75) for nonnormally distributed data and as mean (m) and standard deviation (SD) for normally distributed data. Differences between groups were identified using the Mann–Whitney U test and the Kolmogorov–Smirnov test. Pairwise frequency analysis between groups was performed using chi-squared test and Fisher’s exact test for significance. Correlation scores were determined using the Spearman’s rank correlation coefficient (*p*) and the Pearson linear coefficient (*r*). An alpha level below 0.05 was considered statistically significant (*p* < 0.05).

## 3. Results

### 3.1. Pro- and Anti-Inflammatory Responce of Blood-Derived Monocytes from Patients with Different Durations of T2DM

The basal secretion of TNF-α by blood-derived monocytes from patients with newly diagnosed T2DM was 650.0 (436.8; 922.3) pg/mL in culture medium. IFN-γ-stimulated secretion of TNF-α by cells achieved a value of 1679.5 (1127.5; 2021.5) pg/mL in culture medium (*p* < 0.001). In T2DM patients without DFS with disease duration of 10 years (5; 15), the basal secretion of TNF-α was significantly higher and reached 924.1 (133.8; 1610.0) pg/mL. Following IFN-γ stimulation we found a 1.31-fold increase in the pro-inflammatory response (*p* < 0.001). The basal secretion of pro-inflammatory cytokine in patients with T2DM duration of 12 (6; 20) years and with DFS was lower than in patients without DFS and reached 674.3 (269.3; 1281.1) pg/mL. The stimulated release resulted in a 1.88-fold increase (*p* < 0.001), with no significant difference between the groups. Analysis of anti-inflammatory cell activity by measuring of basal and stimulated anti-inflammatory chemokine (CCL18) secretion levels showed significantly higher chemokine levels in patients with newly diagnosed T2DM compared to patients with long-term diabetes and accounted for 27.5 (14.5; 43.5) pg/mL. Following IL-4 stimulation we observed an increase to 1123.0 (963.2; 1435.2) pg/mL (*p* < 0.001). The basal secretion of CCL18 by monocytes from patients with long-term diabetes without DFS was significantly lower (3.0 (0; 9.6) pg/mL) compared to patients with DFS (9.4 (3.1; 40.1) pg/mL). IL-4 stimulation resulted in a 2.23-fold increase from baseline (*p* < 0.05) in patients without DFS. Induced secretion of CCL18 by monocytes from patients with DFS showed only a 1.36-fold increase (*p* = 0.069) (Table 2).

Thus, activation of the pro-inflammatory response was observed in all patients with different disease durations. In patients with newly diagnosed T2DM, activation of the anti-inflammatory response was maintained; however, there was a significant (*p* < 0.001) decrease in the stimulated secretion of CCL18 in patients with a diabetes duration of more than 10 years.

### 3.2. The Status of Carbohydrate Metabolism and Pro- and Anti-Inflammatory Responce of Blood-Derived Monocytes

In patients with long-term T2DM without DFS, the mean level of HbA1c was 9.0 ± 1.5% and in patients with DFS it was 8.8 ± 1.8%. Differences were not significant (*p* = 0.529). Since the mean age of the patients was 62 years old, in accordance with national clinical guidelines we chose HbA1c ≤ 7.5% as the target level to analyze the status of carbohydrate metabolism. When comparing patients with HbA1c ≤ 7.5% and HbA1c > 7.5%, differences in the basal and stimulated anti-inflammatory response were noted (*p* < 0.05), as shown in Table 3.

Results showed no stimulation of blood-derived monocytes activation to the pro-inflammatory response in patients with DFS with HbA1c ≤ 7.5% and reduced ability to stimulate the anti-inflammatory response, while with decompensated carbohydrate metabolism (HbA1c > 7.5%), the stimulation of the pro-inflammatory activation increased significantly and anti-inflammatory activation increased slightly. In patients without DFS with HbA1c > 7.5%, we found a correlation (*r* = 0.574, *p* = 0.010) between the basal level of TNF-α and HbA1c.

In addition we observed a correlation between HbA1c levels and both TNF-α (*r* = 0.726, *p* = 0.027) and CCL18 (*r* = –0.949, *p* = 0.051) secretion levels by stimulated cells from patients with DFS. Thus, regardless of DFS status, the pro-inflammatory response was not activated in patients achieved the target values of carbohydrate metabolism at the time of examination, whereas it was activated in cases of HbA1c > 7.5%.

### 3.3. Pro- and Anti-Inflammatory Activation Status of Blood-Derived Monocytes from Patients with Lower Limbs Ischemia

**Figure 1:**
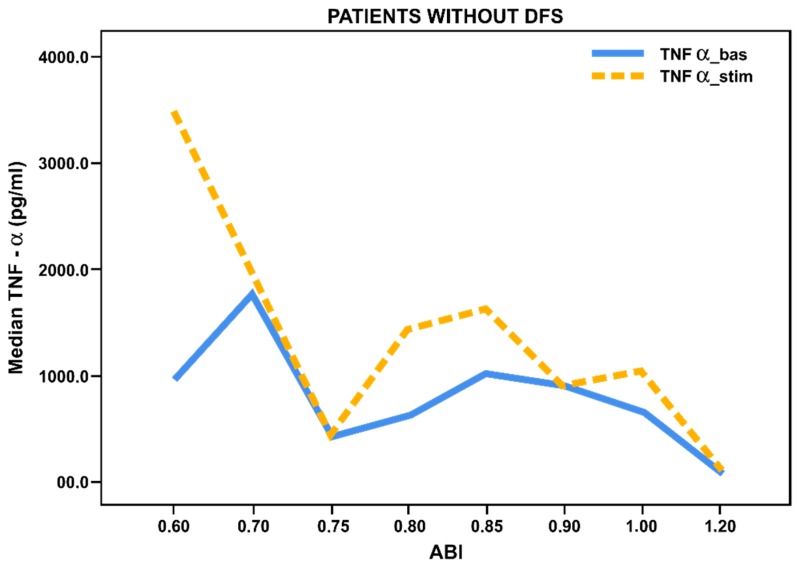
Basal and stimulated TNF-α secretion (pg/mL) by blood-derived monocytes from patients with long-term T2DM (*n* = 51) without foot ulcers, depending on the ankle–brachial index (ABI). Lines reflect median values.

**Figure 2:**
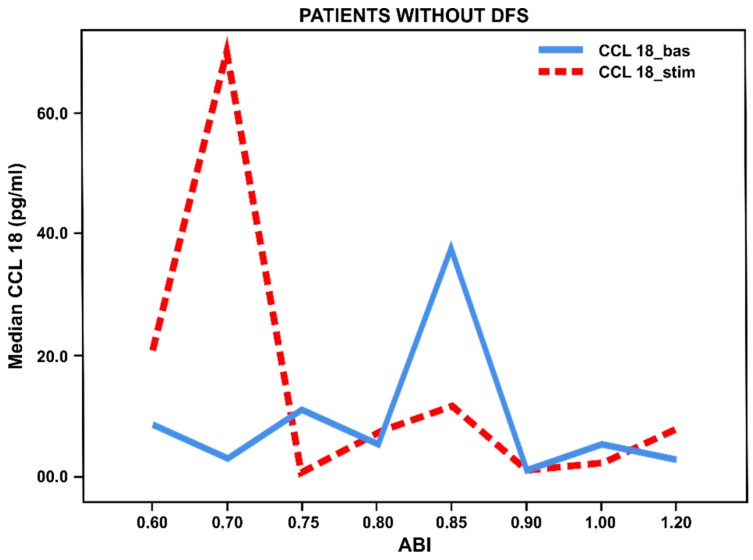
Basal and stimulated CCL18 secretion (pg/mL) by blood-derived monocytes from patients with long-term T2DM (*n* = 51) without foot ulcers, depending on the ankle–brachial index (ABI). Lines reflect median values.

When evaluating ABI values in patients with long-term T2DM without foot ulcers we noted a significant increase in basal and stimulated TNF-α/CCL18 levels in all patients with chronic lower limb ischemia (ABI < 0.8) compared with patients without ischemia (ABI 0.9–1.2) (Figure 1 and Figure 2). It has been found that chronic ischemia of lower limbs in patients without ulcerative defects contributes to activation of blood-derived monocytes to both the pro-inflammatory and anti-inflammatory responses.

**Figure 3:**
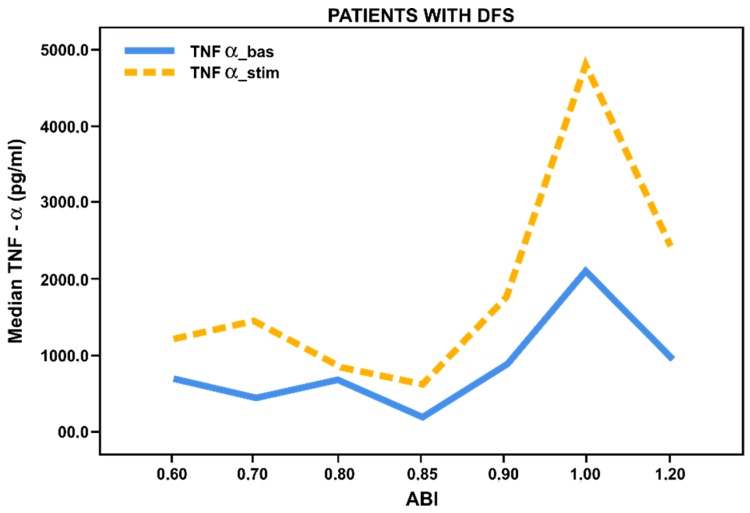
Basal and stimulated TNF-α secretion (pg/mL) by blood-derived monocytes from patients with long-term T2DM (*n* = 42) with foot ulcers, depending on ABI (ankle-brachial index). Lines reflect median values.

**Figure 4:**
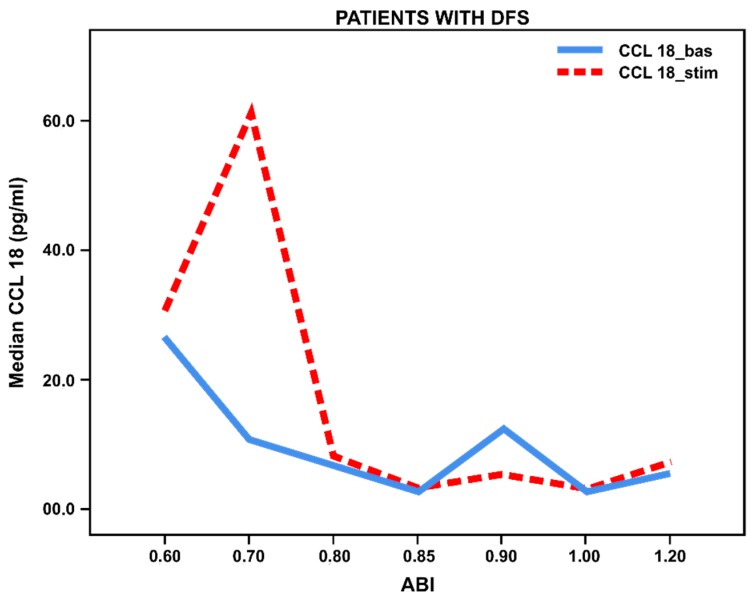
Basal and stimulated ССL18 secretion (pg/mL) by blood-derived monocytes from patients with long-term T2DM (*n* = 42) with foot ulcers, depending on ABI (ankle–brachial index). Lines reflect median values.

However, we observed insignificant secretion of TNF-α and increased CCL18 in patients with foot ulcers and ischemia, while the basal (not stimulated) level of CCL18 was significantly higher in patients with foot ulcers (*p* < 0.027) compared to patients without foot ulcers. Thus, we have found decreased pro-inflammatory response and increased anti-inflammatory activation of monocytes from patients with foot ulcers and ischemia (Figure 3 and Figure 4).

### 3.4. Pro- and Anti-Inflammatory Activation of Blood-Derived Monocytes, Depending on the Duration of the Foot Ulcers

**Figure 5:**
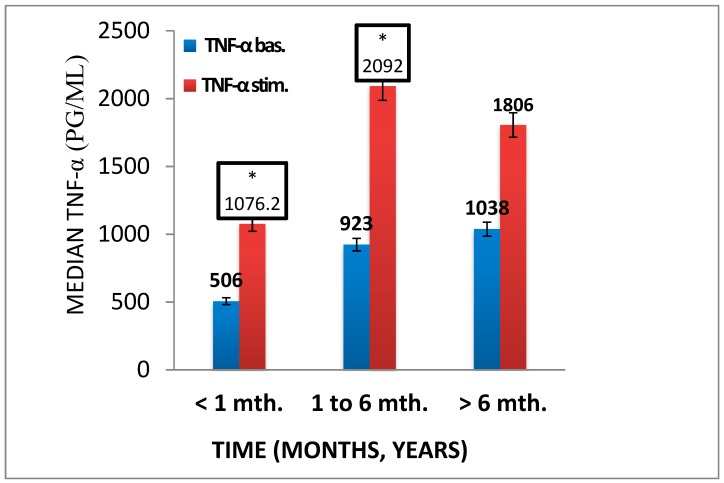
Рro-inflammatory activation cytokine TNF-α in patients with chronic foot ulcers (*n* = 42) of various duration (< 1 to ≥ 6 months (mth). There were 12 patients with less than 1 month of duration, 17 patients with 1 to less than 6 months of duration, and 13 patients with more than 6 months of ulcer duration. Bar histograms display median values, and whiskers reflect IQR (Q25; Q75). Significance level using the Kolmogorov–Smirnov test and Mann–Whitney test, * *p* < 0.05.

**Figure 6:**
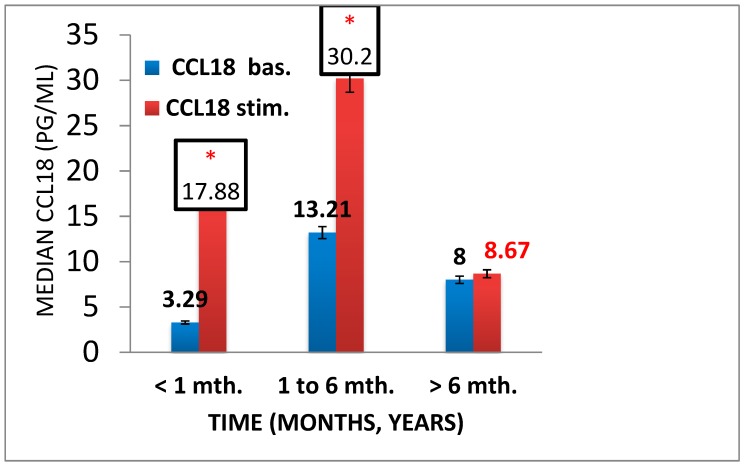
Аnti-inflammatory activation chemokine CCL18 in patients with chronic foot ulcers (*n* = 42) of various durations (< 1 to ≥ 6 months (mth.). There were 12 patients with less than 1 month of duration, 17 patients with 1 to less than 6 months of duration, and 13 patients with more than 6 months of ulcer duration. Bar histograms display median values, and whiskers reflect IQR (Q25; Q75). Significance level using the Kolmogorov–Smirnov test and Mann–Whitney test, * *p* < 0.05.

We also examined monocytes activation in cases of non-healing foot ulcers in patients with T2DM. We have found that throughout the entire inflammatory process, levels of the TNF-α are high with an active stimulation response, *p* < 0.05, whereas during a long-lasting process (more than 6 months) there is no stimulated anti-inflammatory response, *p* = 0.033 (Figure 5 and Figure 6).

## 4. Discussion

According to our previous studies, in patients with newly diagnosed diabetes mellitus who did not receive hypoglycemic therapy there was a significant increased stimulated pro-inflammatory and anti-inflammatory activation of blood-delivered monocytes in comparison with healthy donors [22]. When assessing the activation of monocytes from patients with newly diagnosed T2DM, we have found a more pronounced stimulated pro- and anti-inflammatory response compared to the patients with a long history of the disease. This may be due to the glucotoxicity effect on the monocytes’ ability to polarize to the pro-inflammatory response because of the higher oxidative stress in untreated patients, which involves a significant production of free radicals, non-enzymatic glycated products that lead to the activation of NF-κB that triggers secretion of pro-inflammatory cytokines. [24]

The difference in basal secretion of the pro-inflammatory cytokine between patients without DFS and with DFS did not reach statistical significance (*p* = 0.118). However, the tendency to an increased level of basal TNF secretion by monocytes from patients with type 2 diabetes without DFS may reflect an increased pro-inflammatory activity of monocytes, which may contribute to the gradual formation of a peptic ulcer. The resulting ulcerative defect, in turn, may no longer require the high activity of innate immunity, which is expressed in the relative decrease in basal TNF secretion by monocytes. 

According to our data, patients with untreated T2DM showed a high level of the basal anti-inflammatory response and its significant (approximately 40-fold) activation under IL-4 stimulation. Patients with a long history of the disease showed extremely low activation of monocytes to the anti-inflammatory response as determined by secretion of CCL18 by cultured monocytes. Also, the basal level of CCL18 in patients without DFS was only 3.0 pg/mL, while with IL-4 stimulation it showed moderate activation (2.23-fold), which provides indirect evidence of absence of peripheral inflammation and lack of potential ability to anti-inflammatory response activation in these patients. In contrast, patients with DFS showed a higher basal secretion of CCL18 compared to the patients without DFS, which did not exceed, however, the level of the anti-inflammatory cytokines in patients with newly diagnosed T2DM, thereby indicating the presence of the inflammatory process. However, stimulation of anti-inflammatory activity by IL-4 did not cause the expected adequate activation of monocytes towards anti-inflammatory response monocytes in this category of patients and was even lower (only 1.36-fold) than in patients without DFS (see Table 2). In patients with chronic diabetes, decreased secretion of CCL18 by IL-4-stimulated monocytes was observed compared with a newly diagnosed cohort. Considering such a decrease in ability for induced anti-inflammatory activation of monocytes during a long course of the disease, we regard this as a result of the depletion of the ability of monocytes to form an anti-inflammatory response against a background of chronic inflammation. However, the specific mechanisms responsible for such a decrease remain unclear.

Thus, our ex vivo results are consistent with data in the literature on an increase in pro-inflammatory activity and a decrease in anti-inflammatory activity in in situ diabetic wounds in mice and humans [5,25].

In an attempt to answer the question of why such dysregulation in the activation of monocytes exists in T2DM patients with DFS, we evaluated the effect of carbohydrate metabolism status (compensated/decompensated) on the activation of monocyte responses. The target level of carbohydrate metabolism compensation was HbA1c ≤ 7.5%, in line with national guidelines for patients over 60 years old. Our data have shown no stimulation of activation to the pro-inflammatory response and a decrease of anti-inflammatory response stimulated by IL-4 in patients with DFS with HbA1c ≤ 7.5%. In cases of carbohydrate metabolism decompensation, pro-inflammatory activation of monocytes in patients with DFS increased significantly during stimulation (*p* ˂ 0.001), while activation of the anti-inflammatory reaction was stimulated slightly. We did not observe a stimulation of the pro-inflammatory response in patients with HbA1c ≤ 7.5% without DFS, but we found an active stimulation of the anti-inflammatory response. Such patients with decompensated carbohydrate metabolism (HbA1c ≥ 7.5%) did not show pronounced pro-inflammatory activation nor anti-inflammatory response.

Furthermore, in patients with HbA1c ≥ 7.5% without DFS, a correlation (*r* = 0.574, *p* = 0.010) between the basal levels of TNF-α and HbA1c was found. In cases of foot ulcers, a direct correlation was determined between the level of HbA1c and the stimulated marker of inflammation TNFα (*r* = 0.726, *p* = 0.027), and an inverse correlation was observed between HbA1c and the anti-inflammatory marker CCL18 (*r* = −0.949, *p* = 0.051). Thus, our results have shown a clear relationship between decompensation of carbohydrate metabolism and induction of chronic inflammation in patients with DFS. According to current data, this phenomenon is associated with activation of free radical production and induction of oxidative stress, triggering a cascade of successive events that ultimately lead to imbalanced and dysregulated immune function and prolonged inflammation [26].

It is well established that hypoxia plays an important role in the regulation of wound healing [27]. In this context, we examined the pro-inflammatory and anti-inflammatory reaction activation of blood-derived monocytes from DM patients in relation to the degree of ischemia of the lower limbs. According to our data, patients with foot ulcers and ischemia showed a significant decrease in both basal and stimulated TNF-α production, but in the case of unchanged blood flow in the lower limb vessels we observed a significant stimulation of pro-inflammatory activity that was superior to TNF-α production in patients without foot ulcers. In contrast, anti-inflammatory activation in patients with ischemia and foot ulcers was characterized by less marked stimulated (but not basal) secretion of CCL18, and an abrupt decrease in both the basal and stimulated cytokines in patients with unrestricted blood flow. Such a dysregulation in the pro- and anti-inflammatory activation of monocytes may be associated with a high level of advanced glycoxidation end product (AGE) formation in hyperglycemia settings, which activates oxidative stress, eventually leading to activation of the vascular nitric oxide synthase inhibitor and thereby suppressing the production of NO.

Nitric oxide (NO), a known superoxide scavenger (O^2-^), is the major component of oxidative stress; it stimulates angiogenesis and proliferation through activation of Hypoxia-inducible factors-1 α (HIF-1α) and Vascular endothelial growth factor (VEGF). However, excessive and long-term production of O^2-^ is accompanied by excessive formation of peroxynitrite (ONOO^-^) and peroxynitric acid, which are extremely active prooxidants and significantly inhibit angiogenesis and anti-inflammatory activation of monocytes even in cases of normal peripheral circulation in patients with DFS [28,29].

The development of diabetic foot ulcers is characterized by excessive and prolonged inflammation, leading to poor healing of diabetic ulcers. However, the underlying mechanisms that contribute to excessive inflammation remain poorly understood. The process of wound healing is also associated with the plasticity of monocytes, as they are involved in homeostasis and tissue remodeling [30], since the regenerative potential is associated with monocyte secretion of anti-inflammatory cytokines and growth factors that stimulate the proliferation of fibroblasts. Dysregulation of activation of monocytes is one of the main reasons for delayed wound healing [31,32]. We studied the activation of blood-derived monocytes in patients with different wound healing durations. Our results have shown a significant decrease in the anti-inflammatory response with prolonged duration of the ulcerative defect.

This definitely demonstrates the role of the immune system in the healing process of diabetic foot ulcers and may be associated with effects of hyperglycemia and development of oxidative stress leading to a switch in the mode of monocyte activation and suppression of ulcer healing. Phases of the healing process in patients with DM are also inhibited by other factors, including specific metabolic disorders and impaired physiological reactions such as hypoxia due to hemoglobin glycation, modification of the red blood cell membrane, and vasoconstriction [33].

However, the limitation of current study is the lack of analysis of pro- and anti-inflammatory activation of monocytes in diabetic foot syndrome in parallel in vitro, in vivo, and in situ, which could expand our understanding of the regulation of inflammatory changes in chronic wounds and expand the possibilities of therapeutic approaches to control inflammation in such wounds.

## 5. Conclusions

In conclusion, the analysis of results demonstrates a clear increase in TNF-α and a decrease in CCL18 secretion by blood-derived monocytes from T2DM patients with diabetic foot syndrome in relation to carbohydrate metabolism status (compensated/decompensated) and the severity of ischemia. Our findings allow to us assume that the increase in pro-inflammatory activation and the decrease in anti-inflammatory activation of blood-derived monocytes may cause a slow non-healing chronic wound process.

## Figures and Tables

**Table 1:** Baseline characteristics of the examined patients with T2DM.

Characteristics, Units	1	2	3
	T2DM Newly Diagnosed (*n* = 28)	T2DM without DFS (*n* = 51)	T2DM with DFS (*n* = 42)
Sex, male/female (%)	11(39.3)/17(60.7)	27(52.9)/24(47.1)	29(69.0)/13(31.0)
Age, years	59.1 ± 6.0	62.7 ± 8.2	62.0 ± 8.6
BMI, kg/m^2^	31.5 ± 4.6	32.0 ± 5.0	30.8 ± 5.6
HbA1c,%	9.9 ± 2.3	9.0 ± 1.4	8.8 ± 1.8
Total cholesterol, mmol/L	5.0 ± 1.1	4.9 ± 1.6	5.1 ± 1.4
Triglycerides, mmol/L	2.1 ± 1.4	1.7 ± 0.9	2.1 ± 1.1
HDL-C, mmol/L	1.4 ± 0.6	1.3 ± 0.5	1.4 ± 0.9
LDL-C, mmol/L	3.0 ± 1.5	3.0 ± 1.2	2.9 ± 1.5

Data are presented as mean ± SD and *n* (%). BMI, body mass index; HbA1c, glycated hemoglobin; LDL-C, low density lipoprotein cholesterol; HDL-C, high density lipoprotein cholesterol; T2DM, type 2 diabetes; DFS, diabetic foot syndrome.

**Table 2:** Basal and stimulated TNF-α and СCL18 secretion by blood-derived monocytes from patients with T2DM.

Groups (*n*)	TNF-α pg/mL		CCL18 pg/mL	
	Basal	Stimulated	Basal	Stimulated
(1) T2DM newly diagnosed (*n* = 28)	650.0 (436.8; 922.3)	1679.5 ** (1127.5; 2021.5)	27.5 (14.5; 43.5)	1123.0 ** (963.2; 1435.2)
(2) T2DM 10 (5; 15) years without DFS (*n* = 50)	924.1 (133.8; 1610.0)	1216.0 ** (279.0; 2309.9)	3.0 (0; 9.6)	6.7 ** (1.1; 28.6)
(3) T2DM 12 (6; 20) years with DFS (*n* = 42)	674.3 (269.3; 1281.1)	1271.5 ** (611.9; 2,857.5)	9.4 (3.1; 40.1)	12.8 * (4.1; 67.6)
	(p _1vs2_ = 0.050) * *(p _1vs3_ = 0.606)* *(p _2vs3_ = 0.118)*	*(p _1vs2_ = 0.930)* *(p _1vs3_ = 0.624)* *(p _2vs3_ = 0.640)*	(p_1vs2_ < 0.001) ** (p_1vs3_ < 0.001) ** *(p_2vs3_ = 0.645)*	(p _1vs2_ < 0.001) ** (p _1vs3_ < 0.001) ** *(p _2vs3_ = 0.065)*

Data are expressed as median with IQR (Q25; Q75). T2DM, type 2 diabetes; DFS, diabetic foot syndrome; TNF-α, tumor necrosis factor-α; ССL18, C-C motif chemokine ligand 18. Significance level using the Kolmogorov–Smirnov test and Mann–Whitney test, * *p* < 0.05; ** *p* < 0.001.

**Table 3:** Basal and stimulated TNF-α and СCL18 secretion by blood-derived monocytes from patients with long-term T2DM depending on HbA1c levels.

Parameter, pg/mL	T2DM w/o DFS	T2DM with DFS	*p*-Value	T2DM w/o DFS	T2DM with DFS	*p*-Value
	HbA1c ≤ 7.5%			HbA1c > 7.5%		
TNF-α basal	1610.0 (399.4; 1989.0)	648.0 (261.3; 766.0)	0.258	849.6 (90.2; 1490.5)	828.0 (280.8; 1317.1)	0.790
TNF-α stimulated	1683.0 (536.3; 2737.0) *p* = 0.051	755.0 (526.0; 1053.3) *p* = 0.011 *	0.161	1069.6 (192.0; 2258.9) *p* < 0.001 **	1499.0 (598.9; 3117.5) *p* < 0.001 **	0.135
CCL18 basal	1.9 (0.1; 5.4)	50.0 (2.9; 71.0)	0.014 *	3.5 (0.0; 10.9)	8.0 (3.4; 23.3)	0.013 *
CCL18 stimulated	20.0 (2.7; 115.1) *p* = 0.017 *	8.7 (2.3; 51.4) *p* = 0.594	0.489	2.9 (1.0; 18.4) *p* = 0.024 *	13.1 (4.7; 74.5) *p* = 0.008 *	0.033 *

Data are expressed as median with IQR (Q25; Q75). T2DM, type 2 diabetes; DFS, diabetic foot syndrome, TNF-α, tumor necrosis factor-α; ССL18, C-C motif chemokine ligand 18; w/o, without. Significance level using the Kolmogorov–Smirnov test and Mann–Whitney test, * *p* < 0.05; ** *p* < 0.001.
